# Close of Term at Buffolo Medical College

**Published:** 1855-03

**Authors:** 


*Close of Term at Buffolo Medical College.*—The correspondence below embodies one of those pleasant incidents which occasionally mark the close of a medical term, and we give it publicity in compliance with a resolution of the class requesting us so to do.

Below the correspondence with Prof. White, follow some resolutions personal to ourselves, for the publication of which we render the same apology —the request of the class. The publication in the original department of the Charge to the Graduates has the same excuse. H.

BUFFALO, Feb. 16th, 1855.

PROF. J. P. WHITE:

*Dear Sir,*—At a meeting of the medical class of the University of Buffalo, held Feb. 15th, 1855, W. W. Wynn was called to the chair, and J. Smith chosen secretary. The object of the meeting being stated by the chairman, by the unanimous vote of the class the undersigned were appointed as a committee to solicit you to allow Compton & Gibson, of this city, to engrave a lithographic likeness of yourself, in order that we may take with us or may obtain on our return to our homes, some memento of so faithful and efficient a teacher as yourself, and also that we may hold in our possession a true likeness of the first instructor in obstetrics in the U. S. who has adopted the "*demonstrative*" mode of teaching in that department. In submitting to you the wishes of the class, we have the honor to assure you of their highest regards.

Yours, &c,

ORVILLE R. FLOWER,
ALEX. GRANT,
THOS. MORRISON,
J. T. HUBBARD,
W. HOWELL.

UNIVERSITY OF BUFFALO, Feb. 16, 1855.

GENTLEMEN:

Your letter containing an account of the proceedings of the class had at a meeting held this morming, and conveying their generous request for a lithographic likeness of myself is received.

Next to a clear conscience there is nothing in this world which I am more ambitious to secure than the respect of my medical brethren and pupils. I am, therefore, gratified by this manifestation of attachment on the part of my class. In complying with a wish which I am aware is prompted by their partiality rather than my own merits, permit me to say that I cherish above all price their kind expressions of regard, and highly appreciate their approbation of my efforts in teaching that department of medical science entrusted to my hands by the council of this University.

In the hope that the engraving may be instrumental in perpetuating the good feeling which has uniformly prevailed in the intimate relations which have existed between us for the last few months, and desiring you to convey to the different members of the class whom you represent, the assurance of my affectionate regard,

I remain your friend,

JAMES P. WHITE.

Messrs. ORVILLE R. FLOWER,
ALEX. GRANT,
J. T. HUBBARD,
WM. HOWELL,
THOS. MORRISON,
Committee.

